# Effects of Epimedium supplementation on post-thaw spermatological and oxidative stress parameters in bull semen

**DOI:** 10.1007/s11250-026-05140-4

**Published:** 2026-06-12

**Authors:** Taygun Gökdemir, Nail Tekin Önder, Muhammet Can Kiliç, Oğuzhan Şahi̇n, Lale Başer, Yavuz Öztürkler, Savaş Yıldız

**Affiliations:** 1 Department of Reproduction and Artificial Insemination, Faculty of Veterinary Medicine, Kafkas University, Paşaçayırı, Kars, Turkey; 2 Department of Biochemistry, Faculty of Veterinary Medicine, Kafkas University, Kars, Turkey

**Keywords:** Antioxidant, Epimedium, Spermatozoa, Bull

## Abstract

In this study investigated the impact on Epimedium ***(****Epimedium sp.)* added to the sperm diluent during cryopreservation. For this purpose, semen was collected from Zavot bulls (*n* = 3) using the artificial vagina method and pooled. The pooled semen was diluted with a TRIS-based diluent and divided into four groups. The groups were divided into control and three Epimedium groups (10, 20, 30 µL; E1, E2, E3) and frozen in nitrogen solution vapor (approximately − 120 °C) and sretained in nitrogen solution for evaluation. Following a one-week storage period, the spermatozoa were thawed at 37 °C for 30 s, and sperm motility and kinematics (CASA), plasma membrane integrity (HOST), acrosome integrity (A), mitochondrial membrane potential (MMP), malondialdehyde (MDA) ratio, and glutathione peroxidase (GSH) activities were analysed. It was determined that adding 30 µL of Epimedium to the diluent increased sperm motility and kinematics compared to the control (*p* < 0.05). At the end of the study, Epimedium groups showed more favourable results than the control group in all assessments. Consequently, it was concluded that Epimedium added to the sperm diluent improved spermatological characteristics.

## Introduction

Cryopreservation of semen assists breeders in forming high-quality herds from elite bulls without having to keep the bull on site, thereby reducing costs (Khosla et al. 2022). However, even in the widely accepted protocol for freezing, it has been reported to damage semen (Muino et al. 2007; Nagata et al. 2019). These damages are conditions that put sperm quality and fertility at risk in mammals. For this reason, research is currently on going into adding different substances to the diluent to preserve semen quality during freezing and to develop an effective diluent (Raheja et al. 2018). The beneficial effects of different substances have been reported in different animal breeds (Selvaraju et al. 2016). Studies have shown that natural antioxidants added to semen diluents reduce freezing damage (Gangwar et al. 2018; Ren et al. 2019). Plant polysaccharides are now also being researched as potential antioxidants. Plants possess therapeutic properties such as alkaloids, flavonoids, phenols and terpenes. Along with this diversity, they protect cells from oxidative stress with their antioxidant properties and play a role against anti-inflammatory agents (Sun and Shahrajabian 2023). Plant polysaccharides protect the semen by reducing the damage caused during freezing and thawing by acting as impermeable cryoprotectants, while also increasing antioxidant capacity (Bucak et al. 2007; Hu et al. 2010). Epimedium is a traditional Chinese plant and has been used for over a thousand years (Chen et al. 2014; Xiang et al. 2017). Epimedium is rich in elements such as Zn, Cu, Mn, Se, and Fe, which play a role in sperm formation and morphology and are associated with enzymes in sperm metabolism (Huiling et al. 2000). The osmotic effect of Epimedium polysaccharides leads to dehydration and a reduction in intracellular ice crystal formation. Furthermore, their interactions with phospholipids in the plasma membrane also ensure sperm survival during cryopreservation (Junwei 2014). Epimedium species are rich in flavonoid compounds, particularly icariin and its derivatives, which have been demonstrated to possess strong antioxidant properties. Flavonoids exert their effects by directly scavenging reactive oxygen species (ROS), thereby inhibiting lipid peroxidation and preserving the structural integrity of cell membranes. Spermatozoa are particularly susceptible to oxidative damage due to their high content of polyunsaturated fatty acids. Therefore, the reduction of ROS by flavonoids leads to decreased malondialdehyde (MDA) levels and enhanced glutathione (GSH) activity, ultimately contributing to the maintenance of sperm motility and viability. Furthermore, icariin has been reported to stabilize mitochondrial membrane potential and support ATP production, thereby enhancing sperm motility (Ren et al. 2019).

Therefore, this study aimed to investigate to evaluate whether Epimedium supplementation could improve post-thaw sperm quality by enhancing motility, membrane integrity, mitochondrial activity and antioxidant status.

## Materials and methods

All experimental procedures were approved by the Animal Research Ethics Committee of Kafkas University (KAU-HADYEK/2024 − 195).

### Experimental animals

Three Zavot bulls aged between 2 and 5 years old used at Prof. Dr. Ali Rıza Aksoy Education, Research and Application Farm at Veterinary Faculty of Kafkas University, Kars, Türkiye. Animals kept under the same housing, care and feeding conditions Semen samples were collected from three clinically healthy, fertile Zavot bulls with proven reproductive performance. To minimize individual variability, ejaculates meeting standard quality criteria were pooled prior to experimental processing. Each experimental group was replicated at least ten times. Zavot bulls were selected due to their regional significance and limited population size. This breed is native to the Eastern Anatolia region of Türkiye and represents a locally adapted genetic resource with restricted distribution compared to commercial cattle breeds. The use of this breed allows for the evaluation of cryopreservation strategies in underrepresented and region-specific genetic populations.

### Preparation of semen extender and aqueous extract of epimedium

Tris-citric acid-fructose-egg yolk-glycerol basic diluent was used in the study. Composition of main diluent: A one-step diluent was prepared using THAM [tris(hydroxymethyl)aminomethane] 27.1 g/l, citric acid 14.0 g/l, D-fructose 10.0 g/l, penicillin G 0.3 g/l, dihydrostreptomycin 0.4 g/l, egg yolk 20%, glycerol 6% (Önder at al. 2023). To prepare the epimedium solution, 40 g of epimedium powder was mixed with 800 mL of distilled water. The mixture was centrifuged at 3000 rpm for 10 min, and the resulting solution was filtered through filter paper to obtain the solution. Epimedium extract (*Epimedium sp*.) is commercially produced (business registration number: TR-35-K-000853).

### Semen collection

Semen was collected using the artificial vagina method three times a week (*n* = 10). The collected semen was placed in a 32 °C water bath and brought to the laboratory, where mass activity (≥ 3 out of 0–5) and motility (70%) assessments were performed. Pooling was carried out after the desired values were determined.

### Semen dilution and freezing

The pooled semen was divided into four equally groups and diluted, with one group containing 10 µl (E1), one group containing 20 µl (E2), and one group containing 30 µl (E3) of epimedium. The diluted semen was drawn into 0.25 mL straws and left to equilibrate at 4 °C for 120 min. Following equilibration, the straws were frozen in liquid nitrogen vapour and transferred to liquid nitrogen until examination.

### Semen thawing and evaluation

Frozen semen thawed at 37 °C for 30 s, and motility and kinematic movements, HOS test (HOST), acrosome integrity (A), mitochondrial membrane potential (MMP), malondialdehyde (MDA) ratio, and glutathione (GSH) activities were analysed. Motility and kinematic movements were performed using AndroScope (AndroScope CASA System To Go. Minitube). Hypo-osmotic swelling test (HOST) was determined under a phase-contrast microscope (Nicon Eclipse E400, Japan). HOS test was used to value the functional integrity of the sperm membrane, based on curled and swollen tails. This involved incubating 40 µl of semen with 960 µl of a 100 mOsM hypoosmotic solution (9 g fructose + 4.9 g sodium citrate per liter of distilled water) at 37 ◦C for 60 min. 200 sperm cells were counted and evaluated.

### Flow cytometric analysis

The analysis was performed using Attune NxT Acoustic Focusing Cytometer (Invitrogen, USA). The acrosome integrity was assessed using the fluorescein isothiocyanate–conjugated peanut agglutinin (PNA)/propidium iodide (PI) dual-staining approach. The fluorescence was quantified using a 480 nm excitation wavelengthwith a 10 nm excitation bandwidth. The emitted light was fil-tered using a 530/30 nm filter (BL-1) and a 695/40 nm filter(BL-3). The measurements were recorded using Attune NxTsoftware v2.7 (Thermo Fisher). Following the utilization of forward and side scatter light signals to isolate the cell population, the mean fluorescence intensity of the analyzed spermcells was quantified. The experiment contained a total of 10,000 sperm cells, with a flow rate of 12.5 µL/min. The mitochondrial membrane potential was assessed using Rhodamine 123. Flow cytometric analysis was conducted using the methodology published by Önder et al. (2023).

### Biochemical analysis

Thawed semen samples were subjected to spermatological analyses after centrifugation them at 800 g for 10 min, resulting in the separation of the supernatant. Semen samples were used to measure reduced glutathione (GSH) (Beutler et al. 1963) and malondialdehyde (MDA) (Yoshioka et al. 1979) levels in the biological samples obtained by using spectrophotometer (Epoch, Biotek, USA). As an MDA standard, 1, 1, 3, 3- Tetramethoxypropane was used, and the results were reported as nmol/mL protein. GSH analysis, the samples underwent precipitation using a 10% solution of trichloracetic acid, followed by centrifugation at a speed of 1000 g for 5 min. The reaction mixture consisted of 0.5 mL of semen supernatant, 2 mL of tris hydroxymethyl aminomethane buffer (0.4 M; pH 8.9), and 0.1 mL of l 5,5’-dithio-bis-2-nitrobenzoic acid. The solution was maintained at room temperature for a duration of 5 min, and subsequently measured at a wavelength of 412 nm using the spectrophotometer.

All laboratory procedures involving semen handling, cryogenic materials, and chemical reagents were conducted in accordance with institutional biosafety and laboratory safety guidelines.

### Statistical analysis

Statistical analyses were conducted using IBM SPSS software (SPSS 20.0 Windows - SPSS, Chicago, IL, USA). The Shapiro Wilk test was used to assess for normality. All quantitative data are reported as mean ± standard deviation (SD). No chi-square test was applied in this study; all statistical analyses were performed using one-way ANOVA followed by Tukey’s post hoc test. Statistical significance was determined for P values below 0.05.

## Results

**Fig. 1:**
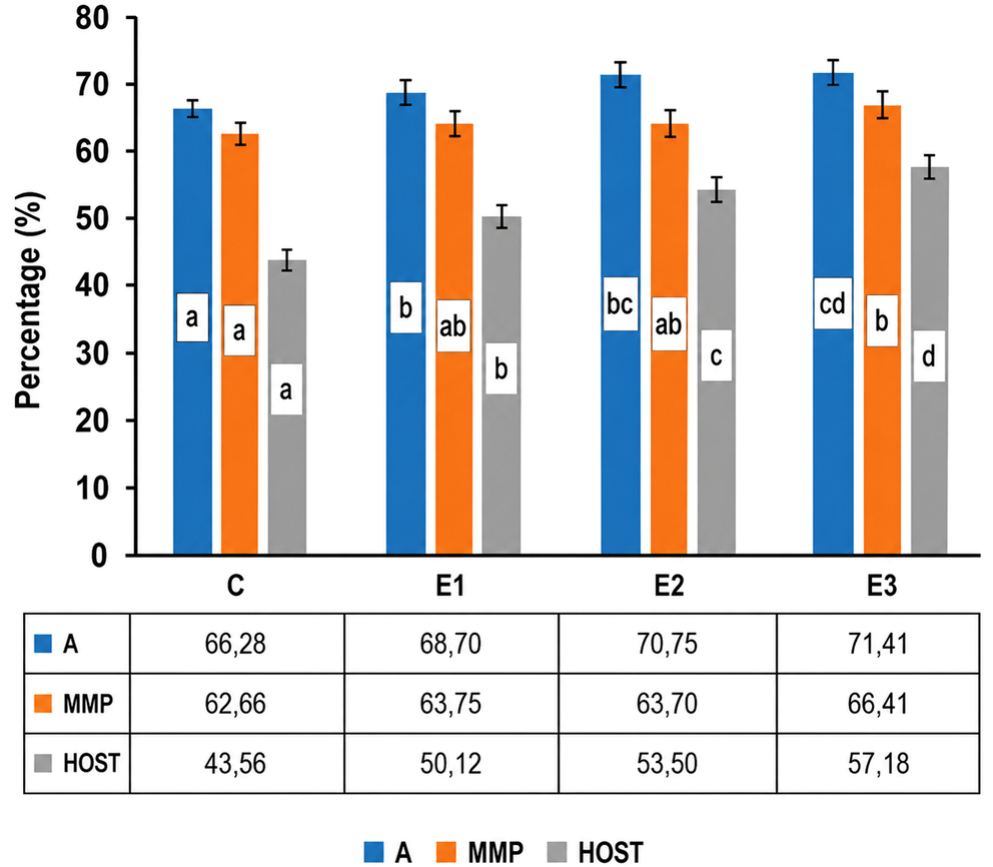
Acrosome integrity, mitochondrial membrane potantial and HOS test in different thawing groups. a, b,c, d: Groups with different letters in the same row and column are statistically different (P < 0.05)

**Fig. 2:**
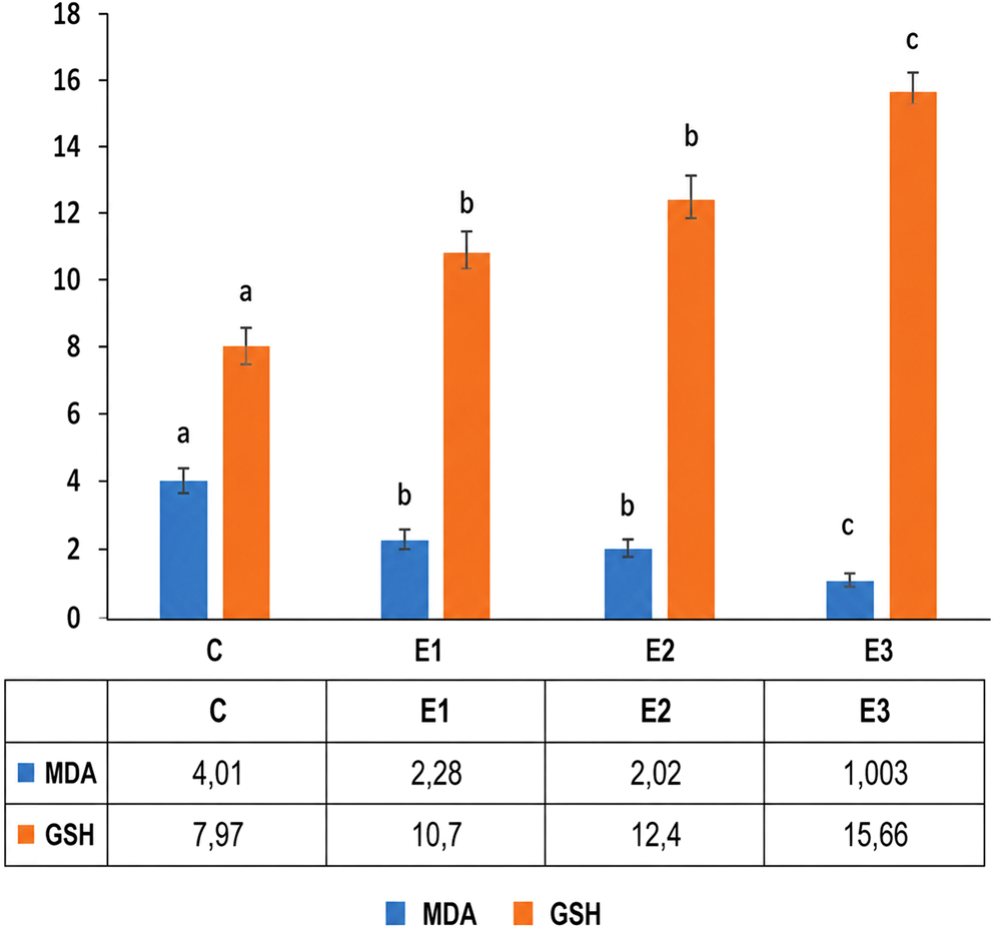
MDA and GSH parameters in different thawing groups. a, b, c: Groups with different letters in the same row and column are statistically different (P < 0.05)

Post-thaw semen motility and kinematic parameters are given in Table 1, acrosome integrity, mmp and hos test results in Fig. 1, and MDA, GSH results in Fig. 2.

As a result of the spermatological evaluations conducted at the end of the research, substantial differences were found between groups in total motility (*p* < 0.05). The highest total motility was recorded in the E3 group (53.50 ± 0.62%), which was significantly higher than all other groups, indicating a dose-dependent effect of Epimedium supplementation. Similarly, progressive motility was significantly increased in the E2 and E3 groups compared to the control group (*p* < 0.05). While there was no difference in progressive motility between groups C and E1 (*p* > 0.05), groups E2 and E3 showed significant differences (*p* < 0.05), with group E3 demonstrating the highest motility. When examining kinematic movements, as seen in Table 1, it is observed that the E3 group significantly improved most parameters. Analysis of kinematic parameters revealed that the E3 group exhibited significantly improved values for VCL, VSL, and VAP, which are indicative of enhanced sperm velocity and forward progression. These parameters are critical indicators of fertilization potential. Looking at A and HOS tests, while E groups showed an increase compared to the control, the greatest difference was again observed in the E3 group. This suggests that Epimedium contributes to the preservation of sperm structural integrity during cryopreservation. For MMP, while there was no difference between groups C, E1, and E2 (*p* > 0.05), E3 yielded different and better results than the control group (*p* < 0.05) (Fig. 1). This finding indicates that higher concentrations of Epimedium are required to exert a measurable effect on mitochondrial function. When evaluating MDA and GSH results, groups containing E showed better results compared to the control, with the E3 group reflecting the most positive data (*p* < 0.05) (Fig. 2). These results confirm the antioxidant role of Epimedium in mitigating oxidative stress during the freeze-thaw process.

Overall, the findings demonstrate that Epimedium supplementation, particularly at the 30 µL level, significantly enhances post-thaw sperm quality through improvements in motility, membrane integrity, mitochondrial function, and oxidative balance.

Improvement was defined as a statistically significant increase in sperm motility, membrane integrity, and antioxidant parameters compared to the control group.

**Table 1:** Motility and kinematic parameters in different thawing groups

Groups	Total Motility (%)	Progressive Motility (%)	VCL (µm/s)	VSL (µm/s)	VAP (µm/s)	DCL (µm)	DSL (µm)	DAP (µm)	ALH (µm)	BCF (Hz)	LIN (VSL/VCL)	STR (VSL/VAP)
C (control)	42.06 ± 0.93^a^	40.16 ± 0.45^a^	75.09 ± 0.39^a^	41.14 ± 0.08^a^	43.83 ± 0.22^a^	30.10 ± 0.05^a^	15.93 ± 0.09^a^	17.08 ± 0.04^a^	1.44 ± 0.01^a^	13.51 ± 0.28^a^	0.51 ± 0.008^a^	0.81 ± 0.01^a^
E1(10 µl Epimedium)	46.70 ± 0.56^b^	42.06 ± 0.35^a^	76.61 ± 0.17^b^	42.93 ± 0.21^b^	45.22 ± 0.24^b^	30.76 ± 0.12^ab^	16.17 ± 0.11^ab^	17.40 ± 0.20^ab^	1.49 ± 0.008^a^	14.01 ± 0.04^ab^	0.54 ± 0.008^a^	0.81 ± 0.008^a^
E2 (20 µl Epimedium)	50.06 ± 0.35^c^	45.50 ± 1.18^b^	78.78 ± 0.37^c^	45.49 ± 0.35^c^	47.25 ± 0.44^c^	31.03 ± 0.08^ab^	16.60 ± 0.20^bc^	17.83 ± 0.12^b^	1.61 ± 0.02^b^	14.80 ± 0.28^b^	0.55 ± 0.005^a^	0.84 ± 0.02^a^
E3 (30 µl Epimedium)	53.50 ± 0.62^d^	49.60 ± 0.28^c^	79.87 ± 0.14^c^	48.75 ± 0.69^d^	48.97 ± 0.08^d^	31.76 ± 0.63^b^	16.96 ± 0.08^c^	17.93 ± 0.03^bc^	1.71 ± 0.01^c^	15.91 ± 0.08^c^	0.59 ± 0.008^b^	0.91 ± 0.01^b^

a, b, c, d: Groups with different letters in the same row and column are statistically different (*P* < 0.05)

## Discussion

Cryopreservation of spermatozoa is associated with multiple stress factors, including cold shock, osmotic imbalance, and excessive generation of reactive oxygen species (ROS), all of which contribute to structural and functional damage in sperm cells. These alterations primarily affect membrane integrity, mitochondrial activity, and ultimately sperm motility and fertilizing capacity (Banday et al. 2017). Due to the limited cytoplasmic antioxidant defense system in spermatozoa, they are particularly susceptible to oxidative stress-induced damage. The biological components of Epimedium (such as flavonoids) are thought to have positive effects due to their antioxidant/membrane protective properties. In the present study, supplementation of the semen extender with Epimedium significantly improved post-thaw sperm quality, particularly at the 30 µL concentration. This improvement was evident in motility, kinematic parameters, membrane integrity, mitochondrial membrane potential, and antioxidant status. The magnitude of improvement observed in the present study was comparable to previously reported antioxidant supplementation studies in bovine semen cryopreservation, particularly regarding motility preservation and membrane integrity. The beneficial effects of Epimedium can be attributed to its rich composition of bioactive compounds, particularly flavonoids such as icariin, which are known for their potent antioxidant properties. These findings are consistent with studies conducted in different animal species. For example, a study by Yaotian et al. (2023) showed that 2 mg/mL Epimedium supplementation increased the fertility of bovine sperm and achieved high artificial insemination pregnancy rates. Although fertility outcomes were not evaluated in the present study, the observed improvements in post-thaw sperm quality parameters may partly explain the increased pregnancy rates previously reported in cattle. Studies in goat sperm (Li et al. 2025) reported that the addition of Epimedium polysaccharides increased post-thaw sperm motility, membrane integrity, acrosome integrity, and total antioxidant capacity. However, the slightly greater improvement observed in bull sperm motility in the present study may be associated with species-specific differences in membrane lipid composition and cryosensitivity. (Despite differences in extract concentration and experimental design, both studies demonstrated that Epimedium-derived compounds reduce oxidative damage during cryopreservation.

**Fig. 3:**
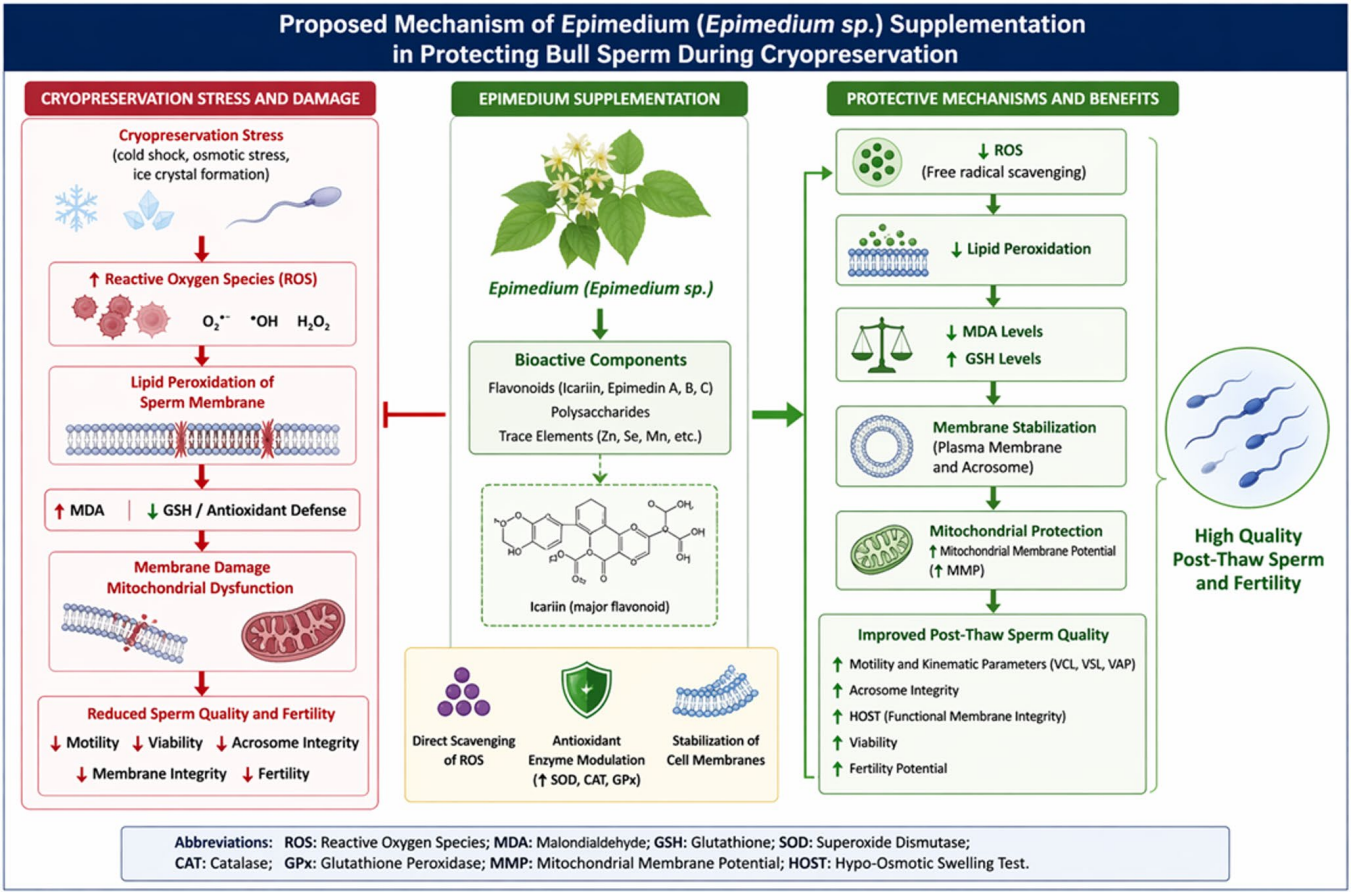
The proposed mechanism underlying the protective effects of Epimedium

Sperm membrane integrity and viability are important factors for sperm quality and function, including the maintenance of homeostasis, ability to move, interact with the environment, and fertilization capability. To achieve these, spermatozoa must have an intact plasma membrane (Hossain et al. 2011). Consequently, there is a relationship between plasma membrane integrity and mitochondrial membrane integrity (Korkmaz et al. 2017). The dense fibrils in the mitochondrial axoneme produce the intracellular ATP necessary for sperm motility (Garnez and Hafez 1993; Bucak et al. 2015). Furthermore, the assessment of mitochondrial membrane potential not only reflects mitochondrial function but also serves as evidence of early apoptosis, as impaired mitochondrial function can lead to increased apoptosis (Paasch et al. 2004). The observed improvements in acrosome integrity and HOST results further indicate that Epimedium plays a crucial role in maintaining sperm structural integrity during the freeze–thaw process. These findings are consistent with previous studies demonstrating that plant-derived antioxidants and Epimedium-derived bioactive compounds enhance post-thaw sperm quality by reducing oxidative stress and preserving membrane integrity (Chen et al. 2014; Ren et al. 2019; Bucak et al. 2007). In agreement with these reports, the present study demonstrated a concentration-dependent improvement, with the 30 µL Epimedium group consistently showing the highest membrane-related protection. Our study found that the addition of epimedium, particularly 30 µl, increased bull sperm acrosome integrity, mitochondrial membrane potential and HOS test ratios. MDA is the end product of lipid peroxidation in the membrane and tends to increase during cryopreservation (Li et al. 2025). The limited size of the spermatozoon cytoplasm results in a low antioxidant reserve, which can lead to the depletion of enzymes such as GSH and the collapse of the defence system due to ROS. Adding antioxidants to the diluent also contributes by reducing the ROS load and improving GSH levels (Karaji et al. 2014). The marked reduction in MDA levels together with increased GSH activity observed in the present study indicates a substantial attenuation of lipid peroxidation during the freeze–thaw process, consistent with previous antioxidant-based cryopreservation studies. In the study, the most significant improvement compared to the control group was observed with the addition of 30 µl of epimedium to the semen diluent (*p* < 0.05). These findings indicate a restoration of the oxidative balance and enhancement of the cellular antioxidant defense system. A study conducted on roosters (Shuyu et al. 2023) reported that epimedium extracts improved semen quality and increased reproductive performance. It was determined that the addition of 0.10% epimedium extract resulted in the highest levels of glutathione peroxidase (GSH-Px) and superoxide dismutase (SOD) activities in the seminal plasma of roosters, while the addition of 0.15% resulted in the lowest levels of malondialdehyde (MDA). The similarity between avian and mammalian findings supports the hypothesis that Epimedium-derived flavonoids exert a broad-spectrum antioxidative effect during cryopreservation. With this consistency these findings collectively suggest that Epimedium may exert similar antioxidative and membrane-protective effects across different species. The proposed mechanism underlying the protective effects of Epimedium during cryopreservation is summarized in Fig. 3.

The use of Zavot bulls in the present study provides valuable insight into the response of a locally adapted and relatively rare cattle breed to cryopreservation protocols. Given the limited distribution and genetic uniqueness of this breed, the findings contribute to the preservation and sustainable use of regional genetic resources.

Taken together, the results of this study suggest that Epimedium exerts a multi-faceted protective effect on spermatozoa by reducing oxidative stress, preserving membrane integrity, and maintaining mitochondrial function. This integrated mechanism ultimately leads to improved post-thaw sperm quality. In conclusion, the present findings suggest that Epimedium supplementation, particularly at 30 µL, may improve post-thaw bull sperm quality by enhancing antioxidant defense and preserving membrane integrity. However, further studies evaluating fertilization outcomes, molecular pathways, and dose optimization are required.

## Data Availability

The data analysed during the present investigation are available from the corresponding author on reasonable request.
